# Perceived risk of technological displacement and mental health among young workers: evidence from the CSS2023

**DOI:** 10.3389/fpsyg.2026.1851198

**Published:** 2026-09-04

**Authors:** Tianfa Yang, Mao Dai, Shuqi Zhang, Ye Guo, Rong Hu

**Affiliations:** 1 School of Public Administration, Guangzhou University, Guangzhou, China; 2 Graduate School of Education, Sehan University, Yeongam-gun, Jeollanam-do, Republic of Korea; 3 School of Education, Guangzhou University, Guangzhou, China; 4 School of Physical Education, Guangzhou University, Guangzhou, China

**Keywords:** mental health, perceived socioeconomic disadvantage, perceived technological displacement risk, social comparison, uncertainty, unemployment expectations

## Abstract

**Introduction:**

This study examines whether perceived risk of technological displacement is associated with the mental health of young workers aged 18 to 35 and explores the mechanisms underlying this relationship.

**Methods:**

Using data from the 2023 Chinese Social Survey (CSS2023) and a nationally representative sample, the analysis employs regression models, heterogeneity tests, and mediation analyses to assess both the overall association and its potential pathways.

**Results:**

The results show that perceived risk of technological displacement is significantly and negatively associated with mental health. This negative association is stronger among workers without higher education, those employed in the market sector, and those living in urban areas. Mediation analyses further indicate that the association is partly accounted for by higher unemployment expectations, greater perceived socioeconomic disadvantage, and more pessimistic expectations regarding family futures.

**Discussion:**

Overall, the findings suggest that perceived technological displacement risk functions as an important psychosocial stressor for young workers and that the mental health consequences of technological change extend beyond objective labor market outcomes to subjective expectations, social comparison, and future-oriented family concerns.

## 1 Introduction

A new wave of technological transformation, driven in particular by generative artificial intelligence (AI), is reshaping labor markets and patterns of skill demand worldwide. Since the emergence of ChatGPT, generative AI has shown a growing capacity to perform cognitive tasks such as text generation, code production, and image creation, extending automation beyond physical labor into a range of knowledge-based activities. In China, the rapid expansion of AI-related industries reflects not only technological growth but also the increasing integration of intelligent systems into economic and organizational life. At the same time, automated decision systems and digital platforms have expanded the reach of technological change well beyond manufacturing, affecting knowledge-intensive sectors such as finance, education, media, and public administration. Against this backdrop, technological change is no longer only an economic issue related to productivity or industrial upgrading; it is also becoming an important source of uncertainty with implications for workers' expectations and psychological wellbeing.

Young workers are both active participants in this wave of technological transformation and direct bearers of the uncertainty it generates. As AI expands into cognitive domains, occupational boundaries and career trajectories are being redefined, intensifying cross-disciplinary competition and increasing the pressure to update skills continuously. At the same time, persistent “involution” (neijuan) has gradually become an implicit institutional norm characterized by relentless and often self-defeating hypercompetition, leaving many young people under sustained pressure from competition and social comparison. In a context of heightened information transparency and increasingly intense public discourse, narratives of “technology replacing humans” further amplify perceptions of risk, transforming a macro-level structural process into an anticipated individual threat and a source of psychological stress. Against this background, an important question arises: is perceived risk of technological displacement associated with the mental health of young workers? If so, what factors may account for this association?

Existing research has mainly examined the consequences of technological change from an objective perspective, focusing on employment restructuring, wage inequality, and differential exposure to automation risk (Autor et al., 2013; Acemoglu and Restrepo, 2020). By comparison, much less is known about how workers subjectively perceive the risk of technological displacement and how these perceptions shape psychological wellbeing. Yet under conditions of rapid technological change, subjective perceptions may matter even before actual displacement occurs. Drawing on the transactional model of stress and coping, perceived threats can generate psychological strain through anticipatory appraisal rather than realized loss alone (Lazarus and Folkman, 1984). This issue may be especially important for young workers, whose career development, status attainment, and family planning are often still in formation. Although a small number of recent studies have begun to examine anxiety related to technological replacement, the psychological mechanisms linking such perceptions to mental health remain insufficiently understood. The present study therefore shifts attention from objective technological shocks to subjective displacement risk and examines how perceived risk of technological displacement is associated with the mental health of young workers. In doing so, it contributes to a more behaviorally informed understanding of the psychosocial consequences of technological change.

This study contributes to the literature in three ways. First, it extends research on technological change by conceptualizing perceived displacement risk as a psychosocial stressor rather than focusing exclusively on objective labor market outcomes. Second, using nationally representative data from the 2023 Chinese Social Survey (CSS2023), it provides micro-level evidence on the association between perceived technological displacement risk and the mental health of young workers, while also identifying differences across educational, employment-sector, and residential groups. Third, it clarifies the mechanisms underlying this association by examining the mediating roles of unemployment expectations, perceived socioeconomic disadvantage, and pessimistic expectations regarding family futures.

## 2 Literature review and theoretical hypotheses

Perceived risk of technological displacement refers to workers' subjective assessment of the likelihood that their jobs may be replaced by artificial intelligence, automation, or robotics (Brougham and Haar, 2020). In the context of rapid advances in AI and automation, technological change has not only restructured employment patterns but also shaped workers' perceptions of risk and their psychological states. Since Keynes (2010) introduced the concept of “technological unemployment,” the relationship between technological progress and labor markets has remained a central concern in both economics and sociology. Building on the longstanding debate on technological unemployment, recent studies have examined how occupational exposure to AI is associated with unemployment risk, job displacement, labor demand, and changing skill requirements (Frank et al., 2025; Acemoglu et al., 2022; Bonfiglioli et al., 2025).

A substantial body of research has shown that objective unemployment has significant negative effects on individual mental health (Gedikli et al., 2023; Farré et al., 2018; Paul and Moser, 2009). However, perceived risk of technological displacement differs from actual unemployment in that it captures workers' forward-looking and subjective assessment of possible future job loss. This distinction is increasingly recognized in recent research on technological change. For example, Hinks (2025) shows that workers' subjective perceptions of threats from AI and other emerging technologies are meaningfully related to their expectations of future wellbeing, underscoring the importance of examining perceived technological risk rather than objective exposure alone. According to the transactional model of stress and coping proposed by Lazarus and Folkman (1984), psychological responses are shaped less by objective events themselves than by individuals' appraisals of potential threats. This suggests that even before technological displacement translates into actual job loss, the uncertainty and stress generated by such perceptions may already damage mental health. Related research also indicates that when individuals perceive their skills or positions to be threatened by machine substitution, they are more likely to experience persistent anxiety, apprehension, and instability in occupational identity, all of which are detrimental to mental health (Tu et al., 2023; Du et al., 2023). On this basis, the study proposes the following hypothesis:

**H1:** Perceived risk of technological displacement is negatively associated with the mental health of young workers.

The effect of perceived risk of technological displacement on mental health may also operate through unemployment expectations. Unemployment expectations reflect workers' forward-looking assessments of their own employment security. When individuals perceive that artificial intelligence or automation may replace their jobs, their expectations of future unemployment are likely to rise accordingly (Chen et al., 2022; Cheng, 2019). Previous studies have shown that stronger unemployment expectations heighten anxiety and psychological pressure, thereby undermining mental health (Suárez-Carreño, 2025; Kopasker et al., 2018; Hannerz et al., 2022). Accordingly, the association between perceived risk of technological displacement and mental health may be partly accounted for by differences in unemployment expectations. On this basis, the study proposes:

**H2:** Higher unemployment expectations mediate the association between perceived technological displacement risk and poorer mental health.

The psychological consequences of perceived risk of technological displacement may also extend to individuals' perceptions of socioeconomic disadvantage. Relative deprivation theory holds that individuals evaluate their position in the social hierarchy through social comparison, and that perceiving oneself as disadvantaged can generate frustration, resentment, and a sense of injustice (Runciman, 1966). Under conditions of rapid AI development, this form of perceived disadvantage may become even more salient. Using CSS2023 data, QQingyi (Zhao and Liu, 2025) found that technological displacement anxiety significantly increases workers' sense of socioeconomic disadvantage. Similarly, Yuzhe (Zhang, 2019) argued that technological shocks may generate not only unemployment risk but also identity anxiety and a diminished sense of self-worth. When young workers perceive machines as having comparative advantages in efficiency or cost, they may interpret this not only as a threat of economic loss but also as a signal of declining social value and relative disadvantage. As perceived socioeconomic disadvantage intensifies, feelings of relative deprivation are likely to become stronger, thereby increasing the risks of anxiety and depression and ultimately undermining mental health. This reasoning leads to the following hypothesis:

**H3:** Greater perceived socioeconomic disadvantage represents a second mediating pathway linking perceived technological displacement risk to poorer mental health.

The consequences of perceived risk of technological displacement may also spill over from the occupational sphere into the family sphere. Beck (1992) argues in Risk Society: toward a New Modernity that modern risks diffuse across social boundaries and extend from the economic sphere into private life. This implies that the psychological consequences of technological displacement are not confined to work-related concerns but may also shape expectations about family life. For young workers, as perceptions of technological displacement accumulate, the occupational uncertainty they generate may gradually erode confidence in household economic stability and future quality of life. Existing studies have shown that unemployment risks associated with technological change affect not only individual income but also family relationships, household expenditure, and living standards (Qiu and He, 2020; Jackson, 2019). At the same time, AI-driven unemployment risks and widening income disparities may weaken individuals' ability to fulfill family responsibilities such as marriage, childrearing, eldercare, and intergenerational support (Adserà, 2025). For young workers who are in the stage of career establishment and family formation, perceived risk of technological displacement may therefore generate not only concern about current income but also pessimism regarding future family arrangements. Accordingly, perceived risk of technological displacement may further undermine mental health by intensifying pessimistic expectations regarding family futures. On this basis, the study proposes:

**H4:** More pessimistic expectations regarding family futures constitute a further mediating pathway linking perceived technological displacement risk to poorer mental health.

**Figure 1:**
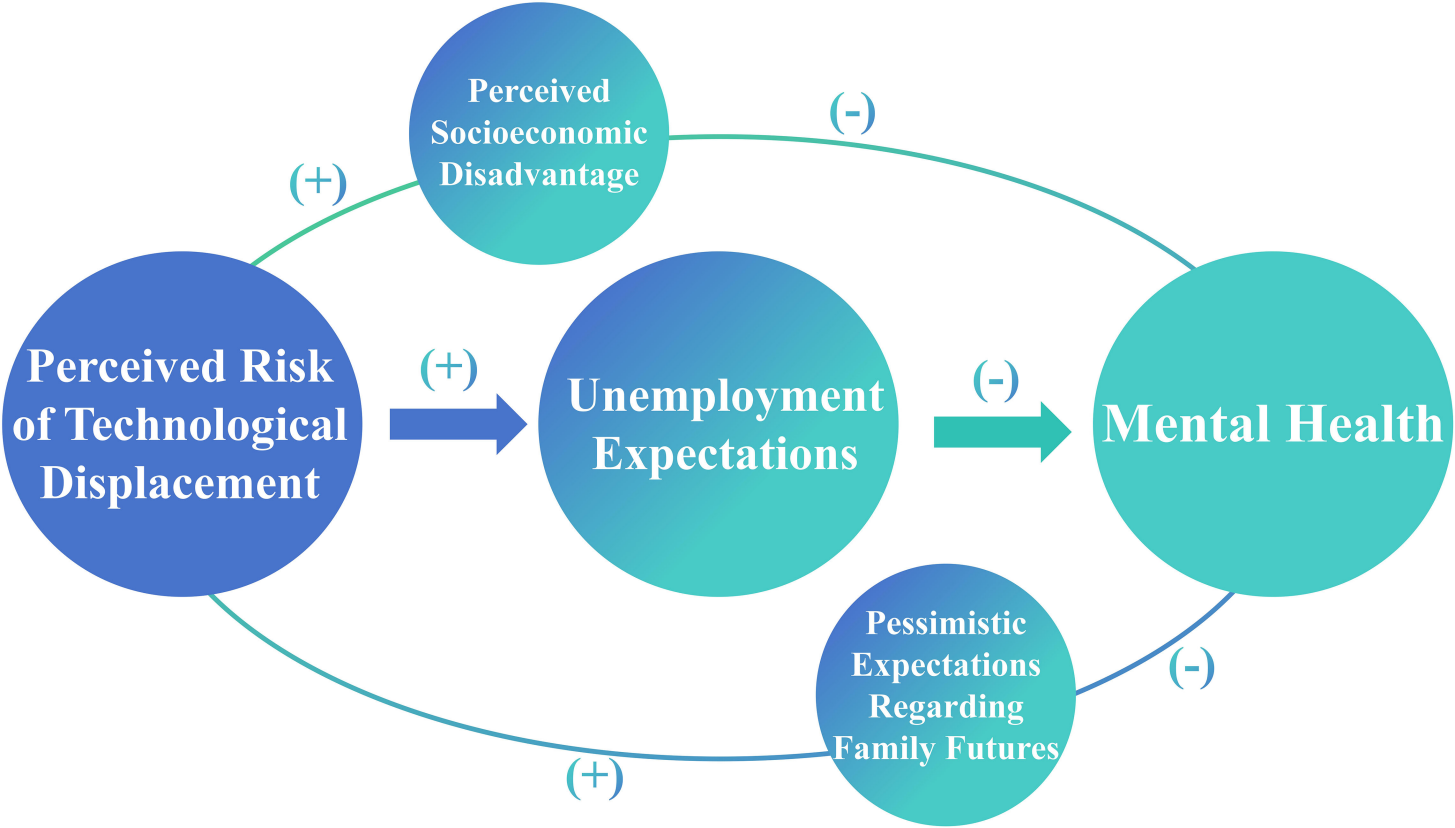
Conceptual framework of the relationship between perceived risk of technological displacement and mental health.

To summarize the theoretical framework and the proposed mechanisms, Figure 1 presents the conceptual model of this study.

## 3 Research data, variables, and model

### 3.1 Data source

This study uses data from the 2023 Chinese Social Survey (CSS2023), a nationally representative survey administered by the Chinese Academy of Social Sciences. The CSS employs a stratified, multistage, probability proportional to size (PPS) sampling design and covers both urban and rural respondents across China. It includes extensive information on demographic characteristics, social attitudes, employment conditions, and mental health, and has been widely used in social science research. A particular advantage of CSS2023 for the present study is that it includes new items on respondents' perceptions of artificial intelligence and the risk of technological displacement, making it possible to examine the subjective experience of anticipated technological replacement and its psychological correlates.

The original CSS2023 dataset contains 13,035 observations. To focus on young workers, this study first selected respondents aged 18 to 35, yielding 3,246 cases. It then excluded respondents who were not currently participating in the labor force, including students, retirees, and the unemployed, reducing the sample to 2,005 observations. Finally, observations with missing values or responses such as “hard to say” or “refused to answer” on key variables were removed. The final analytical sample consists of 1,532 valid cases.

### 3.2 Variable selection

#### 3.2.1 Dependent variable: mental health

Mental health is measured in this study as a self-rated perceptual construct. Following common practice in the public health and health economics literature, mental health is measured using the CSS2023 item: “Overall, how would you assess your current psychological state?” Respondents rate their current psychological state on a scale from one to 10. The variable is treated as continuous, with higher values indicating better mental health (1 = very poor; 10 = very good).

#### 3.2.2 Independent variable: perceived risk of technological displacement

This variable captures the extent to which workers are subjectively concerned that their jobs may be restructured or replaced by technology. It is measured using the item: “What is the likelihood that your job will be replaced by artificial intelligence, robots, or automation technologies?” Responses are coded on a five-point Likert scale ranging from one to five (1 = completely impossible; 5 = completely possible), with higher values indicating greater perceived risk of technological displacement.

#### 3.2.3 Mediating variables

1. Unemployment expectations: this variable reflects individuals' pessimistic assessments of their future employment stability. It is measured using the item: “How likely do you think it is that you will become unemployed within the next 6 months?” Responses range from one to five (1 = completely impossible; 5 = completely possible), with higher values indicating stronger expectations of future unemployment.
2. Perceived socioeconomic disadvantage: this variable captures individuals' subjective perceptions of being socioeconomically disadvantaged relative to others in their local social context. It is measured using the item: “Which level do you consider your current personal socioeconomic status to occupy in your county (county-level city, or district)?” Responses range from one to five. In this study, higher values indicate a stronger perceived sense of socioeconomic disadvantage and, accordingly, stronger feelings of relative deprivation.
3. Pessimistic expectations regarding family futures: this variable reflects the spillover of labor market pressure into the family domain. It is measured using the item: “Thinking about five years from now, do you estimate that your household's economic situation will be much better, somewhat better, the same, somewhat worse, or much worse than it is today?” Responses are coded on a scale from one to five, with higher values indicating greater pessimism regarding future household economic conditions.

#### 3.2.4 Control variables

To reduce potential omitted variable bias, the analysis includes eight control variables at the individual and household levels: gender (male = 1, female = 0), party membership (Chinese Communist Party member = 1, non-member = 0), educational attainment (higher education = 1, no higher education = 0), marital status (married = 1, unmarried = 0), place of residence (urban = 1, rural = 0), sector of employment (in-system sector = 1, market sector = 0), self-rated physical health (Autor et al., 2013; Acemoglu and Restrepo, 2020; Lazarus and Folkman, 1984; Brougham and Haar, 2020; Keynes, 2010; Gedikli et al., 2023; Farré et al., 2018; Paul and Moser, 2009; Tu et al., 2023; Du et al., 2023), and annual income (log-transformed). Taken together, these variables capture demographic characteristics, social resources, and economic conditions that may confound the association between perceived risk of technological displacement and mental health.

### 3.3 Model specification

To examine the proposed statistical pathways, this study employs a stepwise mediation analysis following the Baron and Kenny (1986) framework, complemented by bootstrap tests of indirect associations. Given the cross-sectional nature of the CSS2023 data, the mediation results are interpreted as statistical rather than causal. The analysis assesses whether perceived risk of technological displacement is associated with mental health and whether the proposed mediators account for part of this association. The econometric models are specified as follows:


$$\begin{array}{lll} MHL_{i} & = & \alpha_{0}+\alpha_{1}PTDR_{i}+\beta C_{i}+\varepsilon_{i} \end{array}$$ (1)

$$\begin{array}{lll} MHL_{i} & = & \gamma_{0}+\gamma_{1}PTDR_{i}+\delta C_{i}+\mu_{i} \end{array}$$ (2)

$$\begin{array}{lll} MHL_{i} & = & \omega_{0}+\omega_{1}PTDR_{i}+\omega_{2}M_{i}+\eta C_{i}+v_{i} \end{array}$$ (3)

where *i* denotes the individual; *PTDR*_*i*_ represents perceived risk of technological displacement; *MHL*_*i*_ denotes mental health; *M*_*i*_ refers to the mediating variables, namely unemployment expectations (UE), perceived socioeconomic disadvantage (PSD), and pessimistic expectations regarding family futures (FFP); *C*_*i*_ is the vector of control variables; and ε_*i*_, μ_*i*_, and *v*_*i*_ are stochastic error terms.

Equation 1 estimates the overall association between perceived risk of technological displacement and mental health, which is expected to be negative. Equation 2 estimates the association between perceived risk of technological displacement and each mediating variable. Given the coding of the mediators in this study, perceived risk of technological displacement is expected to be positively associated with each of them. Equation 3 includes both perceived risk of technological displacement and the mediating variable in the mental health equation. If the coefficient of perceived risk of technological displacement decreases in absolute value after the mediator is introduced, and the mediator itself is statistically significant, this provides evidence of a mediation effect. To account for potential overlap among the three mediators, a parallel multiple-mediator model was estimated with all three entered simultaneously into the mental health equation; specific and total indirect effects were assessed via 5,000 bootstrap resamples.

## 4 Results

### 4.1 Descriptive statistics

Table 1 presents descriptive statistics for the main variables. The mean mental health score among young workers is 8.24, indicating a relatively high overall level of mental health. The mean value of the core independent variable, perceived risk of technological displacement, is 2.32, which is below the midpoint of the scale and suggests a generally modest level of perceived risk. At the same time, about 34% of respondents report at least some possibility of technological displacement, indicating that concerns about technology-related job replacement are already salient for a substantial proportion of young workers. With respect to the mediating variables, the mean value of unemployment expectations is 2.30, also below the scale midpoint, suggesting that most respondents are relatively optimistic about their short-term employment prospects. Nevertheless, around 21% report at least some likelihood of becoming unemployed. The mean values of perceived socioeconomic disadvantage and pessimistic expectations regarding family futures are 3.71 and 2.03, respectively. These figures suggest that respondents tend to perceive themselves as moderately socioeconomically disadvantaged while remaining comparatively optimistic about their families' future economic prospects. In terms of sample composition, respondents are predominantly male, married, higher-educated, urban residents, non-party members, and employed in the market sector. Their average monthly income is 6,968 yuan, and the mean level of self-rated physical health is relatively high. Overall, the descriptive statistics suggest substantial variation across both objective socioeconomic characteristics and subjective evaluative dimensions.

**Table 1:** Descriptive statistics.

Variable name	Mean/Percentage	Standard deviation	Minimum	Maximum
Mental health	8.24	1.72	1	10
Perceived risk of technological displacement	2.32	1.21	1	5
Unemployment expectation	2.30	1.25	1	5
Perceived socioeconomic disadvantage	3.71	0.87	1	5
Pessimistic expectations regarding family futures	2.03	0.90	1	5
Physical health level	7.86	1.60	1	10
Male	52.15%	0.50	0	1
Higher education	57.18%	0.49	0	1
CCP member	11.95%	0.32	0	1
Married	56.53%	0.50	0	1
Urban residence	69.84%	0.46	0	1
Employment in the in-system sector	28.79%	0.45	0	1
Annual income (logarithm)	10.93	0.93	4.61	14.29

### 4.2 Baseline regression

Table 2 presents the baseline regression results for the association between perceived risk of technological displacement and the mental health of young workers. Model (1) reports the bivariate association between perceived risk of technological displacement and mental health. Model (2) adds the full set of control variables, and Model (3) further incorporates province fixed effects.

**Table 2:** Full sample benchmark regression results.

Variable	Dependent variable: mental health		
	(1)	(2)	(3)
Perceived risk of technological displacement	−0.204^***^		−0.102^***^
	(0.040)		(0.035)
Physical health level		0.627^***^	0.618^***^
		(0.030)	(0.030)
Male		0.106	0.094
		(0.075)	(0.075)
Higher education		0.053	0.041
		(0.085)	(0.085)
CCP member		−0.014	−0.025
		(0.103)	(0.103)
Married		0.279^***^	0.276^***^
		(0.078)	(0.077)
Urban residence		−0.074	−0.078
		(0.086)	(0.086)
Employment in the in-system sector		−0.010	−0.035
		(0.080)	(0.081)
Annual income		−0.040	−0.050
(Logarithm)		(0.046)	(0.045)
Provinces	NO	Controlled	Controlled
Constant term	8.716^***^	3.326^***^	3.741^***^
	(0.097)	(0.664)	(0.666)
*N*	1,532	1,532	1,532
*R* ^2^	0.021	0.368	0.372
Robust standard errors	YES		

^*^p < 0.10, ^**^p < 0.05, ^***^p < 0.01. Values in parentheses are robust standard errors; the same applies below. ^*^ indicates *p* < 0.10, ^**^ indicates *p* < 0.05, and ^***^ indicates *p* < 0.01.

In Model (1), the coefficient of perceived risk of technological displacement is −0.204 (*p* < 0.01), indicating a significant negative association with mental health in the absence of controls. After the control variables are included, most demographic and institutional factors, including gender, educational attainment, place of residence, party membership, and sector of employment, do not reach statistical significance. By contrast, marital status is positively and significantly associated with mental health, suggesting that married individuals report better mental health than their unmarried counterparts. Self-rated physical health also emerges as an important predictor of mental health: each one-unit increase in physical health is associated with a 0.627-point increase in mental health (*p* < 0.001), underscoring the close relationship between physical and psychological wellbeing.

Most importantly, the results of Model (3) show that perceived risk of technological displacement remains significantly negatively associated with mental health after all control variables are taken into account (β = −0.102, *p* < 0.01). Substantively, this means that each one-unit increase in perceived risk of technological displacement is associated with a 0.102-point decline in mental health. This finding provides support for Hypothesis H1.

### 4.3 Heterogeneity analysis

Given that young workers differ in educational background, sector of employment, and residential context, their resource endowments and capacity to cope with technological displacement may also vary substantially. Accordingly, the association between perceived risk of technological displacement and mental health is likely to be heterogeneous. To examine this possibility, the study conducts a series of subsample analyses, the results of which are reported in Table 3.

**Table 3:** Results of heterogeneity regression analysis.

Variable	Dependent variable: mental health					
	(1) Higher education	(2) Non-higher education	(3) Employment in the in-system sector	(4) Employment in the market sector	(5) Urban residence	(6) Rural residence
Perceived risk of technological displacement	−0.049	−0.163^***^	−0.064	−0.115^***^	−0.117^***^	−0.095^*^
(0.046)	(0.053)	(0.065)	(0.041)	(0.043)	(0.057)
Control variables	Controlled					
Provinces	Controlled					
Constant term	3.737^***^	4.439^***^	2.622^**^	3.931^***^	3.653^***^	4.136^***^
	(0.848)	(0.919)	(1.191)	(0.843)	(0.857)	(1.043)
Sample size	876	656	441	1,091	1,070	462
*R* ^2^	0.405	0.359	0.458	0.360	0.370	0.412
Robust standard errors	YES					

^*^ indicates *p* < 0.10, ^**^ indicates *p* < 0.05, and ^***^ indicates *p* < 0.01.

Heterogeneity by educational attainment: human capital theory suggests that higher educational attainment is generally associated with greater skill transferability, broader employment resources, and stronger adaptability to technological change. Consistent with this expectation, Models (1) and (2) show that perceived risk of technological displacement is not significantly associated with mental health among young workers with higher education, but is significantly and negatively associated with mental health among those without higher education (β = −0.163, *p* < 0.01). This suggests that young workers with weaker educational endowments have fewer resources for occupational transition or skill upgrading and therefore bear greater mental health costs in the face of technological change.

Heterogeneity by sector of employment: labor market segmentation theory argues that different employment sectors are characterized by systematic differences in job security, welfare provision, and occupational risk. In the Chinese context, the distinction between the in-system sector and the market sector reflects differences in institutional protection, job stability, and welfare provision. Jobs in the in-system sector, such as those in government agencies, public institutions, and state-owned enterprises, generally provide stronger employment protection and welfare support, whereas jobs in the market sector are more exposed to market competition and occupational uncertainty. Consistent with this distinction, Models (3) and (4) in Table 3 show that perceived risk of technological displacement is not significantly associated with mental health among young workers employed in the in-system sector, but is significantly and negatively associated with mental health among those employed in the market sector (β = −0.115, *p* < 0.01). This finding suggests that institutional protection partially buffers the psychological impact of technological displacement risk, whereas young workers in the market sector are more vulnerable to its consequences.

Heterogeneity by place of residence: China's longstanding urban-rural dual structure has produced substantial inequalities in employment opportunities, social security, and access to resources, which may in turn shape individuals' ability to cope with technological change. Models (5) and (6) show that perceived risk of technological displacement is negatively associated with mental health in both urban and rural areas. However, the association is stronger among urban residents (β = −0.117, *p* < 0.01) than among rural residents (β = −0.095, *p* < 0.10). This stronger urban association may reflect more intense labor market competition, deeper exposure to technology, and greater pressures of social comparison in urban settings.

**Figure 2:**
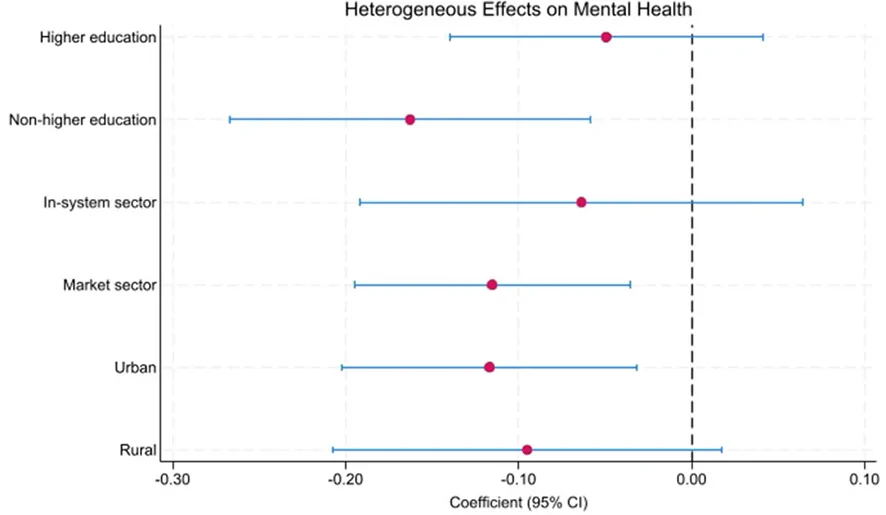
Heterogeneity in the association between perceived technological displacement risk and mental health.

In summary, the negative association between perceived risk of technological displacement and mental health varies systematically across educational attainment, employment sector, and place of residence. Young workers in relatively disadvantaged positions within the labor market appear to exhibit stronger negative associations between perceived technological displacement risk and mental health. To facilitate comparison across subsamples, Figure 2 visualizes the estimated coefficients and confidence intervals for the heterogeneous effects.

The figure highlights the stronger negative associations among less educated workers, those employed in the market sector, and urban residents.

### 4.4 Mediation analysis

The preceding analysis shows that perceived risk of technological displacement is significantly associated with poorer mental health among young workers. Building on this finding, the study examines Hypotheses H2, H3, and H4 by introducing three mediating variables: unemployment expectations, perceived socioeconomic disadvantage, and pessimistic expectations regarding family futures. Table 4 reports the correlations among the three mediators. Given their significant intercorrelations, the study further estimates a joint multiple-mediator model in which all three mediators are entered simultaneously.

**Table 4:** Correlations among the mediating variables.

Variable	(1)	(2)	(3)
Unemployment expectations	1.000		
Pessimistic expectations regarding family futures	0.188^***^	1.000	
Perceived socioeconomic disadvantage	0.240^***^	0.159^***^	1.000
Sample size	1,532		

^*^ indicates *p* < 0.10, ^**^ indicates *p* < 0.05, and ^***^ indicates *p* < 0.01.

Table 5 presents the results of the mediation analysis. The findings show that perceived risk of technological displacement is significantly positively associated with unemployment expectations, perceived socioeconomic disadvantage, and pessimistic expectations regarding family futures. In turn, each of these mediators is significantly associated with poorer mental health, supporting all three proposed pathways. Among them, unemployment expectations account for the largest share of the observed association. This suggests that perceived risk of technological displacement is particularly closely associated with concerns about future employment security. Employment provides not only income but also latent functions such as time structure, social interaction, and occupational identity (Jahoda, 1982). When individuals anticipate possible job loss, they may experience a preemptive sense of deprivation with respect to these functions, which can weaken meaning, reduce self-worth, and intensify psychological stress.

**Table 5:** Mediation effect analysis results.

Variable	Unemployment expectations		Perceived socioeconomic disadvantage		Pessimistic expectations regarding family futures		Joint multiple-mediator model
Dependent variable	MHL	UE	MHL	PSD	MHL	FFP	MHL
Perceived risk of technological displacement	−0.064^*^	0.278^***^	−0.091^***^	0.060^***^	−0.094^***^	0.052^**^	−0.057
	(0.036)	(0.028)	(0.035)	(0.018)	(0.034)	(0.021)	(0.037)
Mediator variable	−0.135^***^		−0.176^***^		−0.143^***^		UE = −0.110^***^
							PSD = −0.143^***^
							FFP = −0.111^**^
	(0.035)		(0.047)		(0.051)		UE: (0.035)
							PSD: (0.047)
							FFP: (0.049)
Control variables	Controlled						
Province fixed effects	Controlled						
Sample size	1,532						
R^2^	0.380	0.229	0.379	0.125	0.378	0.099	0.388
Robust standard errors	Yes						
**Panel B. Separate-model bootstrap indirect effects (5,000 resamples)**							
**Mediator**				**Indirect effect**	**Bootstrap SE**	**Bootstrap 95% CI**	**Proportion mediated**
Unemployment expectations				−0.0375^***^	0.0106	[−0.0591, −0.0177]	36.95%
Perceived socioeconomic disadvantage				−0.0105^***^	0.0043	[−0.0197, −0.0032]	10.34%
Pessimistic expectations regarding family futures				−0.0074^**^	0.0042	[−0.0173, −0.0009]	7.30%
**Panel C. Joint-model specific indirect effects (5,000 resamples)**							
**Mediating pathway**				**Specific indirect effect**		**Bootstrap 95% CI**	
Via unemployment expectations				−0.0305		[−0.0512, −0.0110]	
Via perceived socioeconomic disadvantage				−0.0085		[−0.0168, −0.0021]	
Via pessimistic expectations regarding family futures				−0.0057		[−0.0146, −0.0003]	
Total indirect effect				**−0.0448**		**[−0.0688**, **−0.0233]**	

Bold values indicate the statistically significant total indirect association through all three mediators and its 95% bootstrap confidence interval. ^*^ indicates *p* < 0.10, ^**^ indicates *p* < 0.05, and ^***^ indicates *p* < 0.01.

With respect to perceived socioeconomic disadvantage, young workers who come to believe that their skills may be devalued or rendered obsolete by algorithms or artificial intelligence may develop a stronger sense of relative disadvantage, which may in turn be associated with a weaker sense of social value and less favorable future prospects. This interpretation is consistent with Wilkinson's status anxiety framework, according to which mental health is closely tied to individuals' perceived relative position in the social hierarchy; when people develop a stronger sense of disadvantage, the risks of anxiety and depression may increase accordingly (Wilkinson, 2005). These findings therefore support the proposed statistical mediating role of perceived socioeconomic disadvantage.

Pessimistic expectations regarding family futures also statistically mediate the association between perceived risk of technological displacement and mental health. For young workers who are entering stages of marriage, childrearing, and family responsibility, occupational uncertainty may translate into lower expectations of household income stability, reduced investment in children's education, and weaker capacity for intergenerational support. In this way, perceived risk of technological displacement not only undermines occupational confidence but also erodes expectations regarding future family life. When occupational risk is internalized as long-term family uncertainty, psychological pressure may be further amplified. These results are likewise consistent with the proposed mediating role of pessimistic expectations regarding family futures.

When entered jointly, all three mediators remain significant, while the direct coefficient of perceived risk of technological displacement drops sharply and loses significance. Specific indirect effects through the three pathways are −0.0305, −0.0085, and −0.0057 respectively, with 95% bootstrap confidence intervals all excluding zero; the total indirect effect is −0.0448. This indicates that the three mechanisms, while related, each retain distinct explanatory contributions to mental health.

Bootstrap estimation with 5,000 resamples further confirms this pattern. In the separate mediation models, the three pathways account for 36.95%, 10.34%, and 7.30% of the total association, respectively, with all confidence intervals excluding zero. Taken together, the separate and joint models converge on the same conclusion. Unemployment expectations account for the largest share of the negative association between perceived risk of technological displacement and mental health, while perceived socioeconomic disadvantage and pessimistic expectations regarding family futures account for smaller but statistically significant shares.

### 4.5 Robustness checks

#### 4.5.1 Propensity score matching

Sample selection bias may affect the baseline regression results because perceived risk of technological displacement is unlikely to be randomly distributed among young workers. Instead, such perceptions may be shaped by individual demographic characteristics, socioeconomic conditions, and employment attributes. If this issue is not addressed, the estimated relationship between perceived risk of technological displacement and mental health may be subject to systematic bias. To assess the robustness of the baseline findings, this study employs propensity score matching (PSM), and the results are reported in Table 6.

**Table 6:** Propensity score matching estimation results.

Matching methods	ATT coefficient	S.E.	Significance
Nearest neighbor matching (1:1)	−0.296	0.124	*p* < 0.05
Caliper matching (0.05)	−0.296	0.124	*p* < 0.05
Kernel matching (Epanechnikov)	−0.249	0.097	*p* < 0.01

Respondents were first divided into a treatment group, consisting of those who perceived the risk of technological displacement (*n* = 518), and a control group, consisting of those who did not (*n* = 1, 014). A logit model was then used to estimate each respondent's propensity score, that is, the probability of being assigned to the treatment group, based on gender, marital status, educational attainment, logged annual income, self-rated physical health, place of residence, sector of employment, and party membership. To improve the reliability of the estimates, three matching methods were implemented in parallel: 1:1 nearest-neighbor matching, caliper matching with a caliper width of 0.05, and kernel matching using the Epanechnikov kernel.

The matching results show that all three methods satisfy the common-support condition, and all 1,532 observations fall within the common-support region. This indicates good comparability between the treatment and control groups. The average treatment effect on the treated (ATT) is statistically significant and negative across all three specifications. Specifically, the ATT is −0.296 under both nearest-neighbor matching and caliper matching, and −0.249 under kernel matching. In addition, the standardized bias for all covariates declines substantially after matching, indicating that covariate balance is effectively improved. Taken together, these findings show that, even after observable differences are taken into account, young workers who perceive the risk of technological displacement report significantly lower levels of mental health than those who do not. This result further confirms the robustness of the baseline regression findings.

#### 4.5.2 Alternative coding of the core independent variable and alternative age definition of young workers

To assess whether the findings are sensitive to the operationalization of the core independent variable, this study recodes perceived risk of technological displacement as a binary indicator. Because a response of “neutral” (score = 3) may already reflect a certain degree of risk awareness, responses of three to five are coded as indicating perceived risk of technological displacement, whereas responses of one to two are coded as indicating no perceived risk. The baseline regression and mediation analyses are then re-estimated using this alternative coding scheme. The results show that the direction, statistical significance, and mediation pathways of the key coefficients remain highly consistent with those reported in the baseline model, suggesting that the main findings are not sensitive to the measurement strategy used for the independent variable.

In addition, because the upper age boundary for defining youth remains contested in the literature, this study re-estimates the model using a broader age range of 18 to 45 years, in line with broader international practice (*n* = 2, 930). Under this alternative sample definition, the coefficient of perceived risk of technological displacement remains significantly negative (β = −0.071, *p* < 0.01), which is consistent with the baseline results. Taken together, these findings indicate that the core conclusions of this study are robust to both alternative operationalizations of the independent variable and alternative age-based definitions of young workers.

#### 4.5.3 Sensitivity analysis of the mediation mechanisms

To assess the sensitivity of the mediation results to potential unobserved mediator–outcome confounding, residual-correlation sensitivity analyses were conducted (Imai et al., 2010). The sensitivity parameter ρ represents the correlation between the error terms of the mediator and outcome equations, with ρ = 0 indicating no unobserved confounding.

As shown in Table 7, the estimated indirect effects were reduced to zero only when the residual correlation reached values ranging from −0.090 to −0.109. The pathways through unemployment expectations and perceived socioeconomic disadvantage remained statistically significant under robust inference, while the family-future pathway showed comparatively weaker robustness. Overall, the results provide further support for the stability of the proposed mediation mechanisms.

**Table 7:** Sensitivity analysis of the mediation mechanisms.

Mediator	Indirect effect	Robust 95% CI	ρ at which the indirect effect equals zero	Residual-variance product
Unemployment expectations	−0.0375	[−0.0577, −0.0173]	−0.109	0.0119
Perceived socioeconomic disadvantage	−0.0105	[−0.0188, −0.0022]	−0.105	0.0111
Pessimistic expectations regarding family futures	−0.0074	[−0.0152, 0.0003]	−0.090	0.0081

## 5 Conclusions and discussion

The present findings have important theoretical and practical implications. First, this study extends existing research on technological change by showing that the consequences of AI-related transformation are not limited to objective labor market outcomes such as job loss, occupational restructuring, or wage inequality. Rather, perceived risk of technological displacement itself may function as a meaningful psychosocial stressor associated with poorer mental health among young workers. By shifting attention from objective technological shocks to subjective perceptions of displacement risk, this study contributes to a more behaviorally informed understanding of how technological change shapes individual wellbeing.

Second, the results underscore the importance of examining the psychological mechanisms through which technological anxiety is linked to mental health. The mediating roles of unemployment expectations, perceived socioeconomic disadvantage, and pessimistic expectations regarding family futures suggest that the consequences of technological displacement are not confined to the workplace. The joint multiple-mediator model further indicates that these mechanisms are interrelated but retain distinct explanatory contributions to mental health. Instead, they extend into broader domains of self-evaluation, social comparison, and future life planning. In this sense, the findings add to the growing literature on uncertainty, perceived disadvantage, and mental health by showing that AI-related risk perceptions may operate as a multidimensional source of stress in contemporary labor markets.

Third, the heterogeneity analyses indicate that the psychological burden of technological displacement is unevenly distributed. Young workers with fewer educational resources and those employed in the market sector appear to be especially vulnerable, likely because they have fewer buffers against occupational uncertainty and are more exposed to competitive labor market pressures. From a practical perspective, these findings point to the potential value of targeted mental health screening, career counseling, and skills-based support for groups that are more susceptible to technological uncertainty. Interventions that reduce ambiguity about career prospects, strengthen perceived employability, and enhance coping resources may help mitigate the adverse psychological consequences of perceived displacement risk.

Finally, the findings also point to the importance of social and family-level buffering processes. Because technological displacement anxiety is associated not only with employment-related expectations but also with perceived disadvantage and concerns about family futures, effective responses may need to extend beyond workplace adaptation alone. Psychosocial interventions that address uncertainty management, social comparison, and future-oriented stress may be particularly relevant for young workers navigating rapid technological change. More broadly, this study suggests that understanding the behavioral consequences of AI requires closer attention to how individuals interpret, internalize, and respond to anticipated technological threats in everyday life.

## Data Availability

The data analyzed in this study is subject to the following licenses/restrictions: the data used in this study are from the 2023 Chinese Social Survey (CSS2023), conducted by the Chinese Academy of Social Sciences. Information about the dataset can be found at: http://css.cssn.cn. The data are not publicly downloadable but can be accessed for research purposes upon application and approval from the survey administrators. Requests to access these datasets should be directed to Rong Hu, hurong@gzhu.edu.cn.
